# Stakeholder perceptions of virtual healthcare in Vietnam: qualitative content analysis of doctors, patients, and medical students

**DOI:** 10.1186/s12909-026-10166-1

**Published:** 2026-08-18

**Authors:** Quang Thanh Nguyen, Hoang Viet Nguyen, Ngoc Luong Khanh Nguyen, Lan Thi My Dang, Ha Thi Thu Tran, Jeffrey Mayne, Siaw Cheok Liew, Pranee Liamputtong

**Affiliations:** 1 College of Health Sciences, VinUniversity, Vinhomes Ocean Park, Gia Lam, Hanoi, Vietnam; 2 Department of Surgery, Vietnam National Children’s Hospital, Dong Da, Hanoi, Vietnam; 3 The Royal College of Surgeons in Ireland and University College Dublin Malaysia Campus, George Town, Penang Malaysia; 4 Innovations in Health Sciences, VinUniversity, Gia Lam, Hanoi, Vietnam; 5 Peking University Health Science Center, Haidian District, Beijing, China; 6 Vinmec International Hospital, Times City, Hanoi, Vietnam; 7 University of California, Los Angeles, CA USA; 8 Jeffrey Cheah School of Medicine and Health Sciences, Monash University, Subang Jaya, Selangor Malaysia; 9 College of Arts and Sciences, VinUniversity, Gia Lam, Hanoi, Vietnam; 10 Center for Environmental Intelligence, VinUniversity, Gia Lam, Hanoi, Vietnam

**Keywords:** Virtual healthcare, Virtual consultation, Telemedicine, Medical education, Qualitative survey, Content analysis, Vietnam

## Abstract

**Background:**

Virtual healthcare is increasingly entering clinical practice, yet health professions education has not consistently prepared learners to reconcile technological possibilities with clinical limitations and patient expectations. Multi-stakeholder evidence from lower- and middle-income settings remains limited.

**Methods:**

This qualitative descriptive survey analyzed the free-text component of a broader study using a questionnaire containing structured items and four open-ended questions. Participants were recruited by convenience sampling at a public national pediatric hospital, a private general hospital, and a private not-for-profit medical university in Vietnam from November 2024 to April 2025. Vietnamese doctor and patient or caregiver responses were translated into English and verified against the source text; student responses were collected in English. Two experienced researchers used collaborative consensus coding. Domain presence was recorded once per respondent for each question, and respondent-level percentages were summarized descriptively.

**Results:**

Of 972 parent-survey participants, 952 provided at least one analyzable free-text response. Students had the highest proportion of platform-related descriptions (35.7%) but the lowest proportion referring to clinical care activities (18.8%), compared with doctors (30.8% and 36.5%, respectively) and patients or caregivers (24.7% and 34.0%). Cost, time, and travel saving domain was identified by 66.2% to 80.3% of respondents across groups. Clinical and quality limitations were the leading barrier among doctors (55.2%), whereas technical and workforce barriers were most common among students (54.9%); patient or caregiver responses were divided almost equally between clinical and quality limitations (36.4%) and technical and workforce barriers (35.2%). Appropriate uses centered on common and non-urgent conditions, general advice, mental health care, dermatology and skin conditions, follow-up, and chronic disease management.

**Conclusions:**

Doctors, patients or caregivers, and medical students shared a broad understanding of virtual healthcare but emphasized different aspects of it. These descriptive differences support curricula that connect digital fluency with clinical judgment and patient experience through authentic case selection, remote communication, privacy and consent, explicit recognition of examination limits, and clear escalation to in-person care. The findings are context-specific and should not be interpreted as population prevalence or fixed stakeholder models.

**Supplementary Information:**

The online version contains supplementary material available at https://doi.org/10.1186/s12909-026-10166-1.

## Background

Virtual healthcare refers to health care delivered through digital technologies that support remote interaction, communication, data sharing, self-management, and clinical consultation [1]. Its use accelerated during the COVID-19 pandemic, when telemedicine helped maintain access to non-emergency care in many low- and middle-income countries (LMICs) [2]. Beyond emergency response, virtual healthcare can support more integrated and community-based service delivery in resource-constrained settings [3]. The World Health Assembly has endorsed an extension of the Global Strategy on Digital Health to 2027 [4]. Sustainable implementation, however, requires appropriate governance, infrastructure, and security [5]. Workforce adoption also depends on perceived usefulness, trust, supportive systems, and adequate training [6].

Virtual healthcare is more than a technical innovation. It changes how patients access care, how clinicians assess risk, and how services organize communication and follow-up. Patient experiences are shaped by technical reliability, waiting time, communication quality, and trust [7]. In Vietnam, perceived usefulness, ease of use, perceived risk, and trust in providers have been associated with intention to use telemedicine [8]. The digital determinants of health framework further emphasizes that access to devices, connectivity, and digital literacy influences who can benefit from digital care [9].

These changes have direct implications for medical education. The Association of American Medical Colleges telehealth competencies frame virtual care as a longitudinal clinical capability encompassing patient safety, access and equity, communication, data collection and assessment, technology use, ethical practice, and systems-based care [10]. Educational readiness nevertheless remains uneven. Future physicians commonly report strong interest in telemedicine training but limited academic and practical exposure [11]. Similar gaps in knowledge and experience have been reported among other health professions students [12]. Telehealth learning can strengthen history taking and remote communication, but may restrict physical examination, nonverbal assessment, and learner autonomy when supervision is not well structured [13]. Previous exposure is also associated with stronger intention to use telemedicine in future practice, supporting early and structured integration [14].

Most research examines students, clinicians, or patients separately, even though virtual healthcare depends on interaction among all three groups. Doctors are accountable for diagnostic safety and decisions about escalation. Patients and caregivers experience the practical benefits and burdens of accessing care. Students, particularly those with limited clinical exposure, may initially understand virtual care through the technologies that enable it. Comparing these perspectives can identify where education must connect technological possibilities with clinical limitations and patient expectations rather than assuming that these priorities already align.

Vietnam provides an important setting for this inquiry. Hospital-based telehealth initiatives have connected provincial services with higher-level expertise and supported remote examination and treatment [15]. The 2023 Law on Medical Examination and Treatment formally recognizes remote medical examination and treatment [16], and Circular 30/2023/TT-BYT specifies diseases and health conditions eligible for telemedicine [17]. Despite this policy development, the extent to which virtual healthcare is systematically integrated into undergraduate medical education remains poorly documented. Research from Vietnamese medical education indicates that learners’ acceptance of educational technologies is shaped by perceived usefulness, socioeconomic context, and educational background [18]. Regional evidence likewise identifies digital literacy and perceived usefulness as important influences on telemedicine adoption [19]. Reviews of digital health education emphasize practical application, faculty development, and alignment with local needs [20].

This study aimed to explore how doctors, patients or caregivers, and medical students in Vietnam define virtual healthcare, perceive its benefits and barriers, and identify health issues they consider appropriate for virtual care. By examining the three perspectives together, the study sought to inform locally relevant health professions education and implementation while remaining attentive to the descriptive depth of free-text survey data.

## Methods

### Study design and setting

This qualitative survey study formed part of a broader project examining the knowledge, attitudes, and practices of multiple stakeholders regarding virtual consultation. The Vinmec and VinUniversity Institutional Review Board approved the broader project on August 22, 2024 (approval number 86/2024/QD-VMEC), before recruitment and data collection began in November 2024. Electronic informed consent was obtained before participants could proceed with the survey.

The parent survey used a validated questionnaire containing structured items and four open-ended questions. The present analysis focused only on the free-text responses: (1) “In your view, what does virtual healthcare mean?”; (2) “What are the benefits of virtual healthcare?”; (3) “What are the barriers to the implementation or use of virtual healthcare?”; (4) “What kind of health issues would you use virtual healthcare for?”. The doctor and patient or caregiver versions were administered in Vietnamese, whereas the medical student version was administered in English. The wording above is the English wording used with students and the translation used for reporting. At the pediatric recruitment site, the patient or caregiver versions of questions 2 and 4 referred specifically to benefits for the respondent and their child and to the child’s health issues. Patient or caregiver responses to these questions are therefore not fully equivalent to the unrestricted versions administered to doctor, student, and general-hospital patient respondents.

Guided by a qualitative descriptive approach within a pragmatic orientation, we used qualitative content analysis to describe recurring meanings and compare stakeholder emphases. Frequency summaries supported interpretation but were not used to infer prevalence in the wider population or test causal hypotheses. Reporting was guided by the Standards for Reporting Qualitative Research (SRQR) [21]. Normalization Process Theory (NPT) was used only as a sensitizing lens in the Discussion to consider how shared meaning, participation, collective work, and appraisal may influence the normalization of virtual healthcare; it did not determine the codebook or primary analysis [22].

### Participants and recruitment

Participants were recruited by non-probability convenience sampling through Vietnam National Children’s Hospital, a public national pediatric referral hospital; Vinmec International Hospital, a private general hospital; and VinUniversity, a private not-for-profit medical university. The parent sample comprised 370 doctors (198 from the public pediatric hospital and 172 from the private general hospital), 382 patient or caregiver respondents (205 and 177, respectively), and 220 medical students. The Microsoft Forms survey link was made available through participating sites between November 2024 and April 2025. Doctors were practicing clinicians, patient-group respondents were patients, service users, or caregivers recruited in hospital settings, and medical students were enrolled at the participating university.

A centralized invitation denominator was not retained; therefore, parent-survey response rates and nonresponse characteristics could not be calculated. Detailed characteristics such as physician specialty and years of practice, patient age and education, student year of training, and previous virtual-healthcare experience were not retained in the qualitative extract. This limits assessment of transferability and is acknowledged below.

### Translation and data preparation

Responses were exported from Microsoft Forms. Before coding, blank entries, punctuation-only strings, “N/A,” uninterpretable strings, and explicit statements of no view or no knowledge without substantive content were excluded for the relevant question. Brief but meaningful answers, including single-word responses such as “privacy” or “mental health,” were retained. The number of analyzable responses was calculated for each question and stakeholder group. Response length was summarized as the median and interquartile range of whitespace-delimited words among analyzable responses.

Responses submitted in Vietnamese were translated into English before collaborative coding so that both coders could work from a common analytic language. During coding, a Vietnamese-speaking researcher checked each English rendering against the original response, and ambiguous words or phrases were resolved through coder consensus before a code was assigned. Responses originally submitted in English were retained in their original language. This procedure constituted translation verification. Because the survey was anonymous, participants were not recontacted. Quotations were labeled with anonymized respondent identifiers and recruitment context.

### Research team and reflexivity

Two researchers conducted the analysis: one medically trained researcher with an MD and one researcher with a PhD, both experienced in qualitative research. Coding was collaborative and consensus-based from the outset rather than independently scored and subsequently reconciled. The coders jointly reviewed responses, developed preliminary codes, refined operational definitions, and resolved ambiguous cases through discussion. The MD researcher contributed familiarity with clinical language and service delivery, while the PhD researcher contributed qualitative and interpretive expertise. The team recognized that clinical experience could increase sensitivity to safety and diagnostic concerns; iterative discussion was used to keep interpretations close to participants’ wording. An audit trail preserved source responses, translations, coding decisions, category definitions, and analytic outputs.

### Qualitative content analysis

The analysis proceeded in three stages. First, the coders read the responses to each question and developed descriptive codes inductively from recurring words, phrases, and semantic meanings. Second, related codes were grouped into broader domains with explicit operational definitions. For question 1, “virtual consultation” captured the interactional mode of remote advice or consultation, whereas “clinical care activities” required explicit reference to diagnosis, treatment, prescribing, monitoring, or another clinical function; responses could receive both codes when both elements were present. Third, the coded dataset was restructured at the response level so that each domain was recorded as present or absent once per respondent for each question. A response could contribute to more than one domain, but repeated references to the same domain within one response were counted once. The final domains summarized major recurring patterns and were not forced to exhaust every meaningful response; responses that did not fit a displayed domain remained unclassified. An illustrative coding progression and the complete domain matrix are provided in Additional file 1.

R version 4.5.2 was used only after coding to calculate response-completeness and word-count summaries, aggregate the consensus response-level domain indicators, calculate the mean number of displayed domain assignments per analyzable response, and produce the figures [23]. The figures report the percentage of analyzable respondents within each stakeholder group who mentioned each domain at least once. No inferential tests were conducted because the study was designed as descriptive qualitative research and did not specify confirmatory between-group hypotheses. Because coding was collaborative from the outset, no interrater reliability coefficient was calculated. Additional file 1 reports the coding framework, domain-assignment intensity, and the minimum number of analyzable responses not represented by a displayed domain.

### Trustworthiness

Trustworthiness was supported through collaborative interpretation by two analysts with complementary clinical and qualitative expertise, comparison of three stakeholder data sources, maintenance of an audit trail, explicit operational definitions, translation verification against the Vietnamese source, and presentation of contrasting stakeholder quotations. Frequency summaries were used as a descriptive complement to, rather than a substitute for, qualitative interpretation.

## Results

### Participant contribution and response characteristics

Of the 972 parent-survey participants, 952 (97.9%) provided at least one analyzable free-text response (Table 1). Question-level completion remained high overall, although it was lower among patient or caregiver and student respondents than among doctors (Table 2). Responses were brief, with overall median lengths of 6 to 10 words per question. The findings below describe patterns among respondents who supplied analyzable text for each question; they should not be interpreted as population prevalence or statistically tested group differences.

**Table 1:** Participant groups, recruitment context, and contribution to the qualitative analysis

Stakeholder group	Recruitment context and site distribution	Parent survey, *N*	At least 1 analyzable response, *n*/*N* (%)
Doctors	Public national pediatric referral hospital, *n* = 198; private general hospital, *n* = 172	370	370/370 (100%)
Patients/service users/caregivers	Public national pediatric referral hospital, *n* = 205; private general hospital, *n* = 177	382	366/382 (95.8%)
Medical students	Private not-for-profit medical university, *n* = 220	220	216/220 (98.2%)
Total	—	972	952/972 (97.9%)

**Table 2:** Question-level response completeness and response length. Completion percentages use the parent-survey count in each stakeholder group as the denominator. Word counts are median (interquartile range) among analyzable responses

Question	Doctors	Patients/service users/caregivers	Medical students	Overall
Q1: Meaning	370/370 (100%) 11 (6–18.8) words	356/382 (93.2%) 9 (5–17) words	213/220 (96.8%) 8 (4–15) words	939/972 (96.6%) 10 (5–17) words
Q2: Benefits	370/370 (100%) 8 (5–14) words	352/382 (92.1%) 8 (5–14) words	213/220 (96.8%) 6 (3–14) words	935/972 (96.2%) 7 (4–14) words
Q3: Barriers	368/370 (99.5%) 11 (6–18.3) words	341/382 (89.3%) 8 (4–16) words	206/220 (93.6%) 8 (4–17) words	915/972 (94.1%) 9 (5–17) words
Q4: Appropriate uses	367/370 (99.2%) 7 (4–12) words	355/382 (92.9%) 6 (3–11) words	205/220 (93.2%) 4 (2–11) words	927/972 (95.4%) 6 (3–12) words

Mean displayed domain assignments per analyzable response ranged from 0.97 to 1.79 across stakeholder-question cells (Additional file 1, Table A3). Because the final domains were non-exhaustive, some meaningful responses did not map to a displayed domain; aggregate counts imply a minimum of two patient or caregiver responses for question 3 and nine for question 4 without a displayed-domain assignment. Cross-group domain percentages therefore reflect response elaboration as well as thematic emphasis

### Perceived meanings and uses of virtual healthcare

**Fig. 1:**
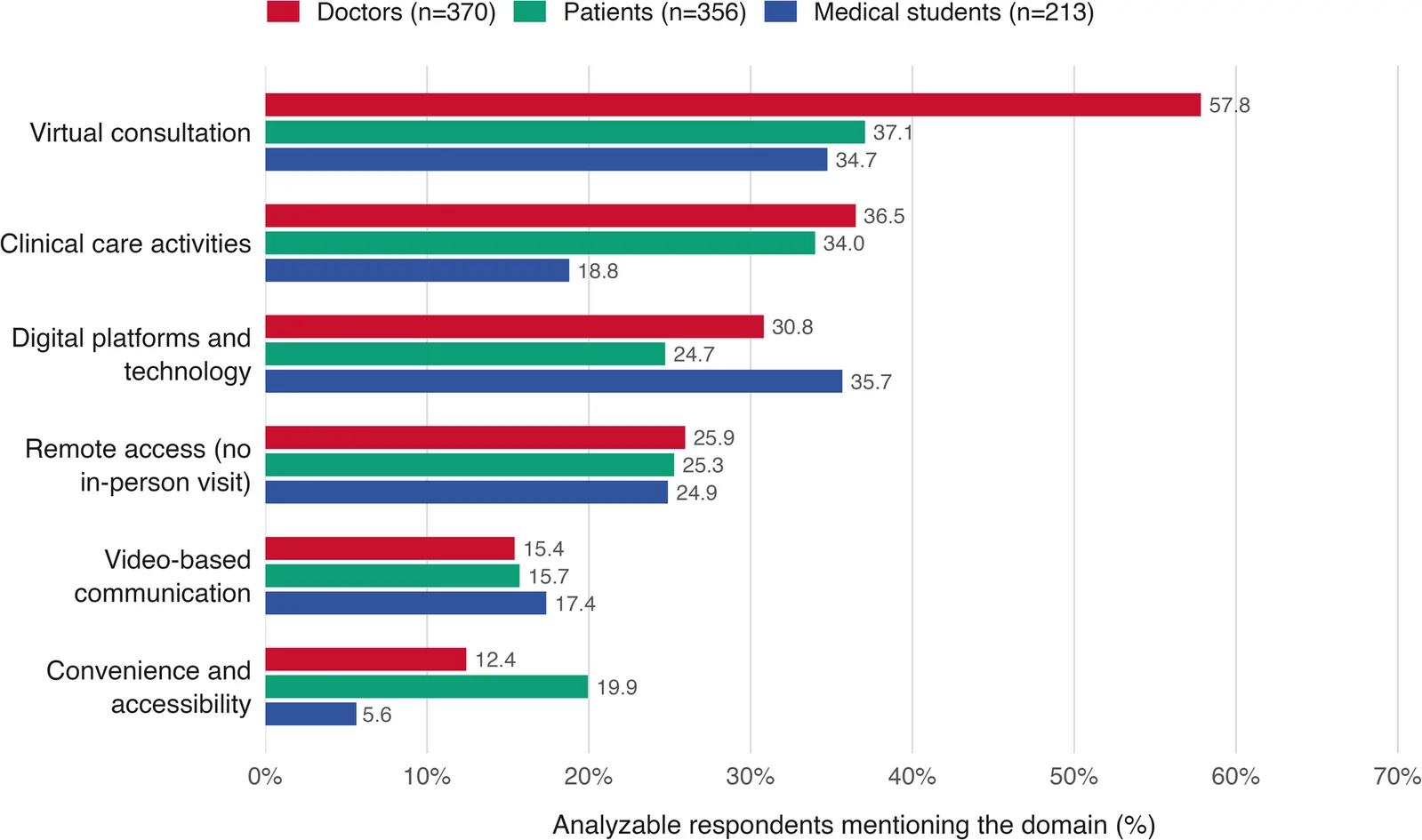
Perceived meanings and uses of virtual healthcare across stakeholder groups. Bars show the percentage of analyzable respondents within each stakeholder group who mentioned each domain at least once. Q1 denominators were 370 doctors, 356 patients or caregivers, and 213 medical students. Responses could contribute to multiple domains, and the final domains were non-exhaustive; percentages may therefore sum to more or less than 100%

The three groups shared a broad association between virtual healthcare, remote interaction, digital platforms, and consultation, but the distribution of domains differed descriptively (Fig. 1). Medical students had the highest proportion of platform-related descriptions (35.7%, 76/213) but the lowest proportion referring to clinical care activities (18.8%, 40/213). Corresponding proportions were 30.8% (114/370) and 36.5% (135/370) among doctors and 24.7% (88/356) and 34.0% (121/356) among patient or caregiver respondents. Virtual consultation was identified by 57.8% (214/370) of doctors, 37.1% (132/356) of patient or caregiver respondents, and 34.7% (74/213) of students. Convenience and accessibility appeared in 19.9% (71/356) of patient or caregiver responses, compared with 12.4% (46/370) of doctor responses and 5.6% (12/213) of student responses.

“Virtual healthcare is consultation, diagnosis, and health monitoring delivered remotely through technology platforms such as websites, mobile applications, or video calls. It allows patients to reach doctors without going directly to a hospital or clinic.” [DNCH128, doctor, public pediatric hospital; translated from Vietnamese]

“Virtual healthcare means receiving online advice from doctors when needed, especially when a family member has a health problem and does not need to go to the hospital immediately.” [PVMEC91, patient/caregiver, private general hospital; translated from Vietnamese]

“Virtual healthcare refers to the delivery of healthcare services through digital platforms and technology, allowing patients to consult with healthcare providers remotely rather than through in-person visits. This can include video consultations, remote monitoring, telemedicine, mobile health apps, and online health records… It often relies on technologies like video conferencing, wearable health devices, and AI-driven health assessments.” [SVUNI83, medical student; original English]

### Perceived benefits of virtual healthcare

**Fig. 2:**
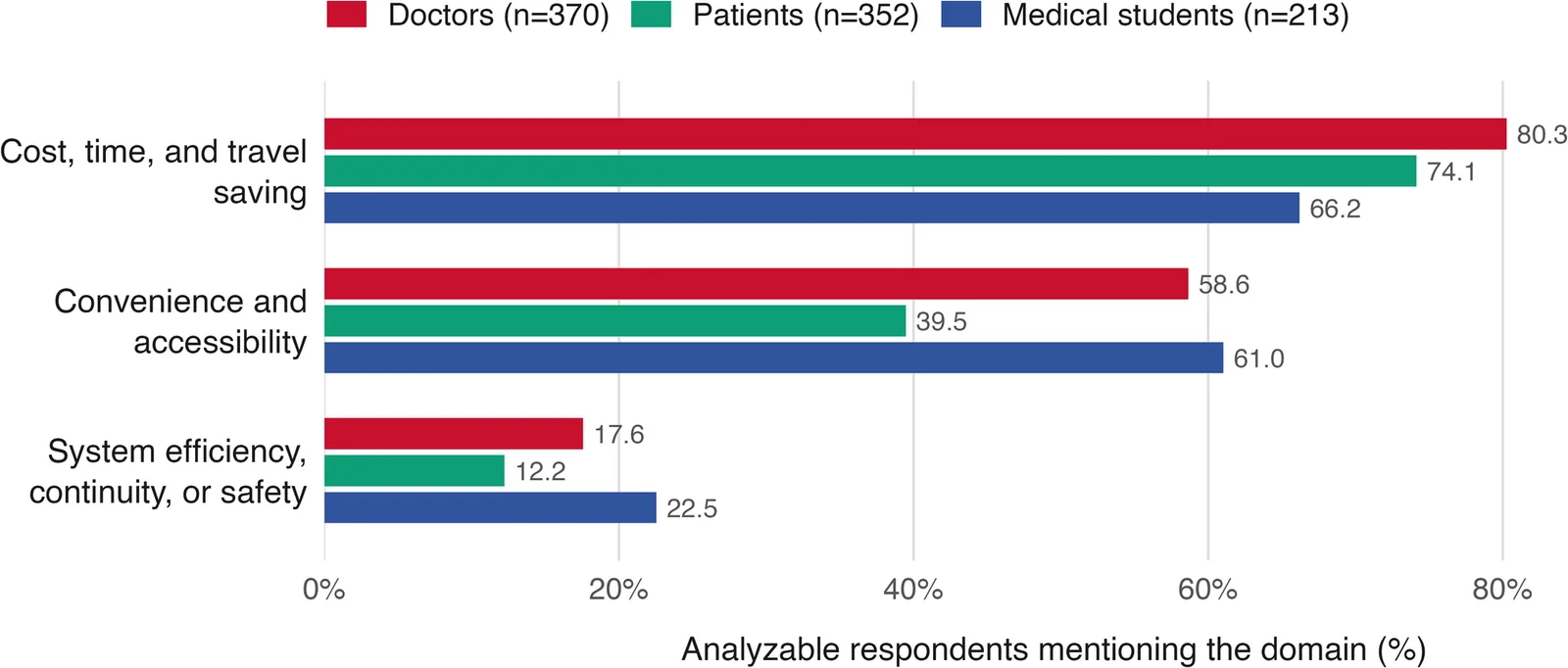
Perceived benefits of virtual healthcare across stakeholder groups. Bars show the percentage of analyzable respondents within each stakeholder group who mentioned each domain at least once. Q2 denominators were 370 doctors, 352 patients or caregivers, and 213 medical students. Responses could contribute to multiple domains, and the final domains were non-exhaustive; percentages may therefore sum to more or less than 100%. At the pediatric hospital, the patient or caregiver question referred specifically to benefits for the respondent and child

Cost, time, and travel saving was the most consistently reported benefit, identified by 80.3% (297/370) of doctors, 74.1% (261/352) of patient or caregiver respondents, and 66.2% (141/213) of students (Fig. 2). Convenience and accessibility were also prominent, particularly among students (61.0%, 130/213) and doctors (58.6%, 217/370), compared with 39.5% (139/352) of patient or caregiver respondents. System efficiency, continuity, or safety appeared less often in doctor (17.6%, 65/370), patient or caregiver (12.2%, 43/352), and student responses (22.5%, 48/213). Within this system-level domain, explicit references to care coordination were uncommon.

“Virtual healthcare saves patients time and travel costs, helps people in remote areas access health services, reduces pressure on hospitals and clinics, supports continuous health monitoring, especially for patients with chronic disease, and limits the risk of infection in health facilities.” [DNCH128, doctor, public pediatric hospital; translated from Vietnamese]

“Patients and family members would not have to miss school or work, arrive early, or queue for a number.” [PNCH82, patient/caregiver, public pediatric hospital; translated from Vietnamese]

### Perceived barriers to implementation

**Fig. 3:**
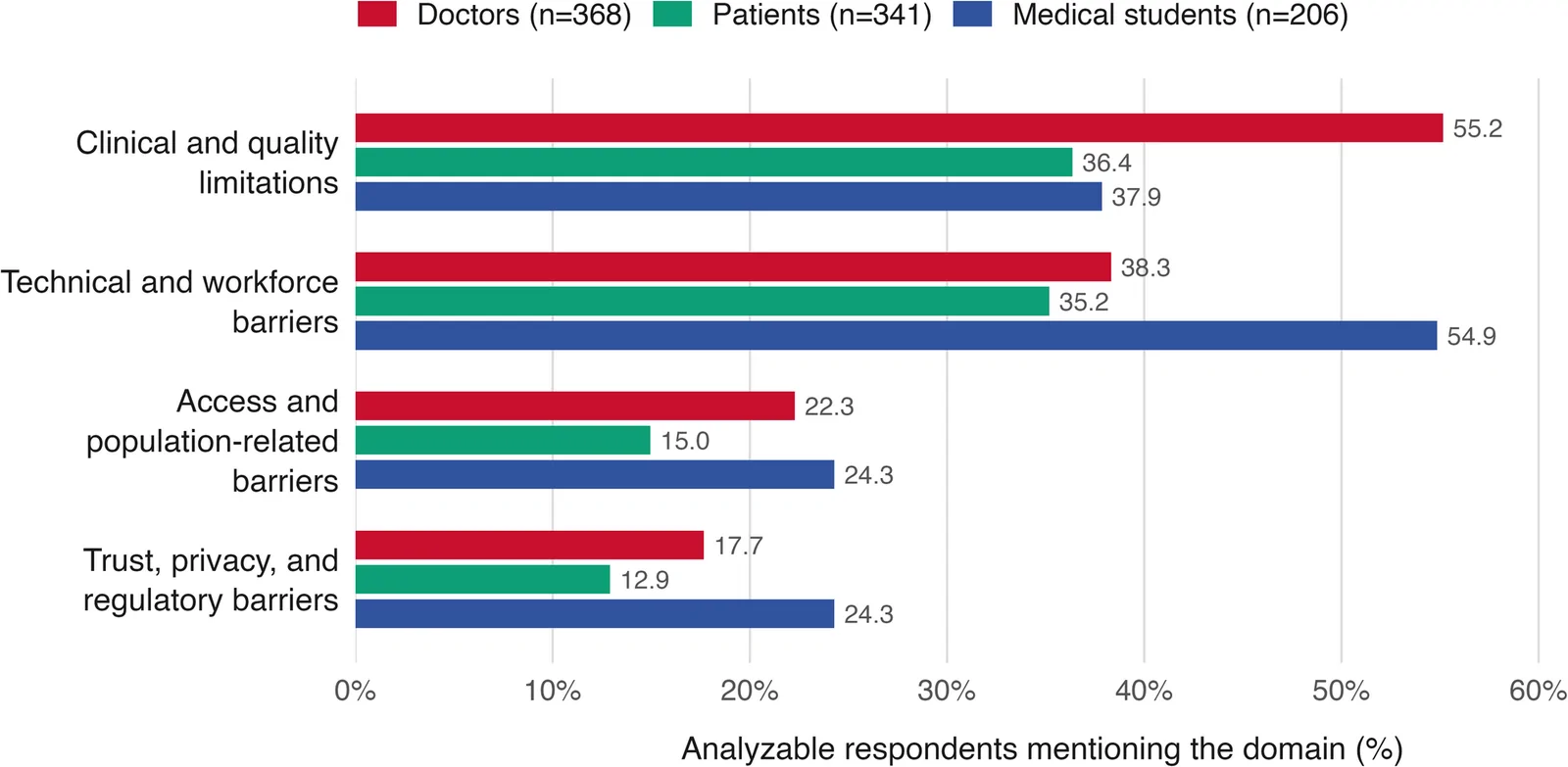
Perceived barriers to virtual healthcare implementation across stakeholder groups. Bars show the percentage of analyzable respondents within each stakeholder group who mentioned each domain at least once. Q3 denominators were 368 doctors, 341 patients or caregivers, and 206 medical students. Responses could contribute to multiple domains, and the final domains were non-exhaustive; percentages may therefore sum to more or less than 100%

Among doctors, clinical and quality limitations were identified by 55.2% (203/368), followed by technical and workforce barriers (38.3%, 141/368), access and population-related barriers (22.3%, 82/368), and trust, privacy, and regulatory barriers (17.7%, 65/368). Among students, technical and workforce barriers were most frequent (54.9%, 113/206), followed by clinical and quality limitations (37.9%, 78/206); access and population-related barriers and trust, privacy, and regulatory barriers were each identified by 24.3% (50/206) (Fig. 3). Among patient or caregiver respondents, clinical and quality limitations (36.4%, 124/341) and technical and workforce barriers (35.2%, 120/341) were reported almost equally often, followed by access and population-related barriers (15.0%, 51/341) and trust, privacy, and regulatory barriers (12.9%, 44/341).

“Online services reach only people with smart devices and an internet connection, which limits access in remote areas with poor connectivity. Diagnosis is also constrained because assessment is limited to questioning, listening, and visual observation.” [DNCH12, doctor, public pediatric hospital; translated from Vietnamese]

“As a medical student, I see several barriers to implementing virtual healthcare. One significant challenge is the lack of reliable internet access in rural areas, limiting its reach to underserved populations. Additionally, many patients, especially older ones, are not familiar with using technology, creating a digital literacy gap. There’s also the issue of trust—some patients may doubt the accuracy of virtual diagnoses compared to in-person consultations. Finally, ensuring data privacy and security is a major concern, as sensitive medical information must be protected in online platforms.” [SVUNI149, medical student; original English]

“I worry about scams, such as making an appointment and then no doctor appearing. I would feel more reassured if the service were provided by a hospital. This is still new in Vietnam, and people may not be used to it. I still feel safer with an in-person examination because even direct examinations can be wrong, and online examinations may be even less certain.” [PVMEC25, patient/caregiver, private general hospital; translated from Vietnamese]

### Health conditions considered appropriate for virtual healthcare

**Fig. 4:**
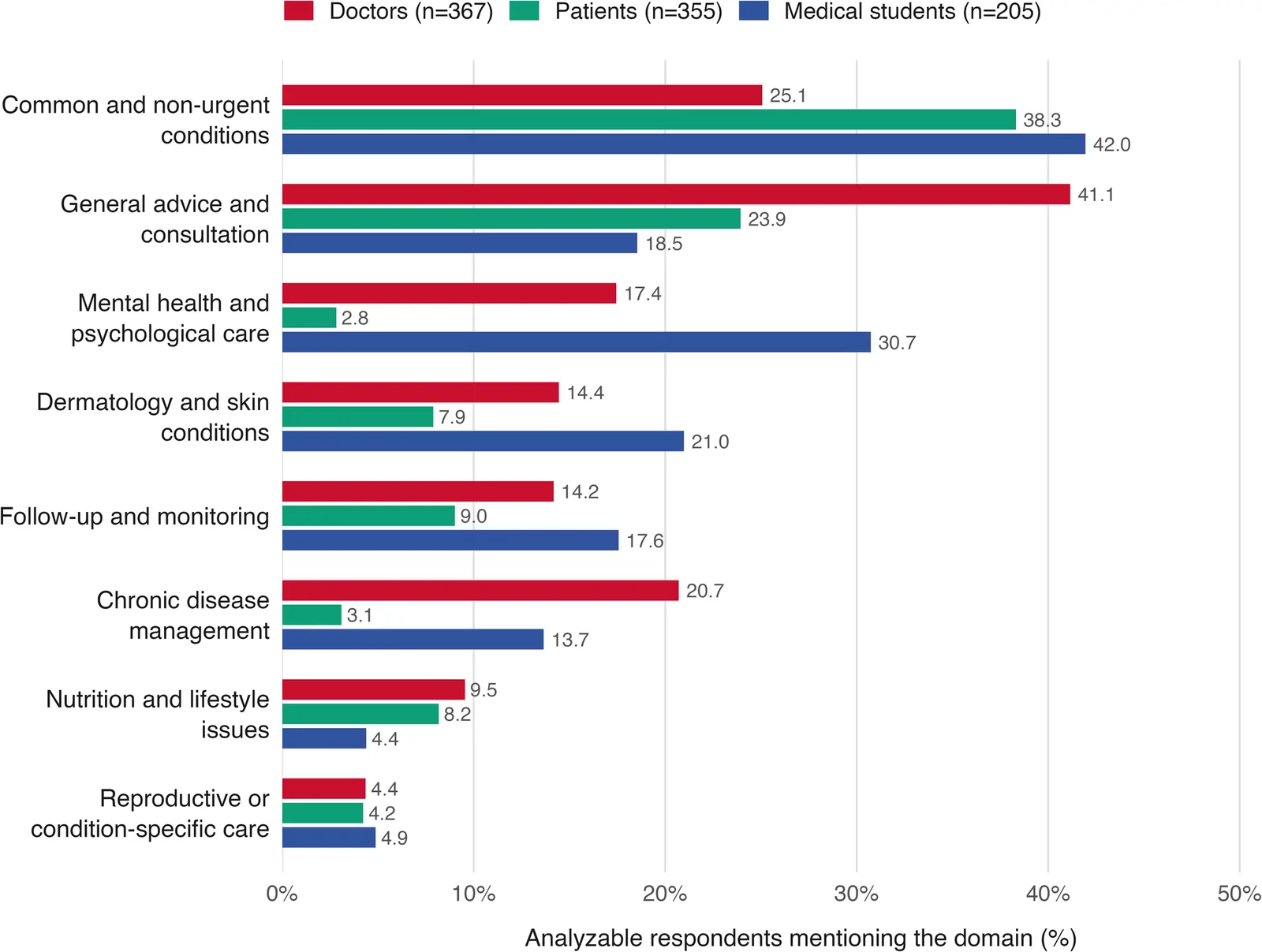
Health conditions considered appropriate for virtual healthcare across stakeholder groups. Bars show the percentage of analyzable respondents within each stakeholder group who mentioned each domain at least once. Q4 denominators were 367 doctors, 355 patients or caregivers, and 205 medical students. Responses could contribute to multiple domains, and the final domains were non-exhaustive; percentages may therefore sum to more or less than 100%. At the pediatric hospital, the patient or caregiver question referred specifically to the child’s health issues

Appropriate-use responses centered on common and non-urgent conditions, general advice, mental health care, dermatology and skin conditions, follow-up, and chronic disease management (Fig. 4). General advice and consultation were identified by 41.1% (151/367) of doctors, while 25.1% (92/367) identified common and non-urgent conditions. Among patient or caregiver respondents, 38.3% (136/355) identified common and non-urgent conditions and 23.9% (85/355) identified general advice and consultation. Students identified common and non-urgent conditions (42.0%, 86/205), mental health and psychological care (30.7%, 63/205), dermatology and skin conditions (21.0%, 43/205), follow-up and monitoring (17.6%, 36/205), and chronic disease management (13.7%, 28/205). Chronic disease management was identified by 20.7% (76/367) of doctors but by 3.1% (11/355) of patient or caregiver respondents.

“Suitable uses include periodic health checks; monitoring healthy people and patients with stable chronic disease who do not require emergency care; and managing common infectious illnesses without warning signs.” [DVMEC64, doctor, private general hospital; translated from Vietnamese]

“When a child has a mild cough or a minor oral problem, medication may be prescribed without complex tests.” [PNCH65, patient/caregiver, public pediatric hospital; translated from Vietnamese]

“I would use virtual healthcare for non-emergency health issues such as follow-up consultations, managing chronic conditions like diabetes or hypertension, mental health therapy, routine check-ups, and minor ailments like colds, flu, or skin rashes… Virtual healthcare is ideal for conditions that do not require physical exams or immediate in-person treatment.” [SVUNI83, medical student; original English]

## Discussion

This qualitative survey identified shared recognition of virtual healthcare as digitally mediated care alongside different stakeholder emphases. Students had the highest proportion of platform-related descriptions but were less likely than doctors or patient and caregiver respondents to describe virtual healthcare in terms of clinical care activities. Doctors more often emphasized consultation and clinical functions. Patient and caregiver respondents more often used convenience and accessibility when defining virtual healthcare in question 1, but they did not report convenience or cost, time, and travel benefits more frequently than the other groups in question 2. Because the student questionnaire was administered in English whereas doctor and patient or caregiver responses were collected in Vietnamese and translated, the platform-versus-clinical contrast may partly reflect language and academic register as well as stakeholder role. The brief responses therefore support descriptive differences that may create mismatched expectations, not deep or fixed conceptual models.

NPT offers a useful sensitizing lens for these findings [22]. Differing descriptions suggest incomplete shared sense-making, or coherence, across stakeholder groups. The extent to which clinicians, patients, caregivers, and learners regard virtual care as legitimate and worth supporting relates to cognitive participation. Concerns about examination, connectivity, training, privacy, and workflow relate to the collective work required for implementation, while judgments about benefits and appropriate cases resemble reflexive monitoring. This interpretation remains provisional because NPT was not used prospectively and the survey cannot establish how normalization occurs within specific organizations.

Practical benefits were prominent across groups, with 66.2% to 80.3% of respondents to the benefits question mentioning savings in cost, time, or travel. Evidence from LMICs similarly emphasizes improved access and reduced disruption to care [2]. Patient research also highlights convenience and reduced travel burden [7]. Research involving clinicians and patients shows that these benefits can coexist with concerns about clinical evaluation and communication [24]. System-level evidence identifies potential gains in information sharing, efficiency, and referral coordination, while noting that many digital initiatives remain fragmented or poorly aligned with local services [25]. The low salience of explicit care-coordination references within the system-level benefit domain may indicate that respondents encountered virtual healthcare mainly as individual consultations rather than as part of an integrated pathway. This interpretation is tentative because the survey did not examine actual service organization or patterns of use.

Clinical adequacy was a central concern among doctors, whereas students more often identified technical and workforce barriers. Telemedical consultation research similarly identifies limited physical examination, reduced overall clinical impression, infrastructure problems, and technical failure as recurring constraints [26]. Organizational research shows that training, confidence, and institutional support influence clinician acceptance [27]. Patient studies emphasize communication quality and technical reliability [7], while research with medical students shows that reduced visual cues and limited physical examination can constrain learning [13]. The educational response should therefore extend beyond platform training. Students need practice determining which information can be obtained remotely, recognizing diagnostic uncertainty, communicating limitations, and escalating to in-person care. Patient-derived cases should also demonstrate how convenience, caregiving burden, connectivity, and digital literacy affect whether a technically available service is genuinely accessible.

The respondent-level analysis also helps interpret the comparatively low frequency of chronic disease management among patient or caregiver respondents. Chronic disease management was identified by 20.7% of doctors and 13.7% of students who answered the appropriate-use question, but by 3.1% of patient or caregiver respondents. Vietnam’s Law on Medical Examination and Treatment provides a legal basis for remote medical examination and treatment [16], and the national telemedicine circular identifies diseases and health conditions that may be managed remotely [17]. The lower patient or caregiver frequency may partly reflect the pediatric recruitment context, child-referenced wording at that site, and the shorter responses and lower mean number of displayed domain assignments in the patient or caregiver group. However, participants’ chronic disease status and prior experience with remote chronic care were not collected, so the study cannot establish why this domain was less salient or attribute the pattern to national insurance or hospital workflows.

These findings support a direct educational response to the stakeholder differences. The Association of American Medical Colleges (AAMC) telehealth competency domains provide a practical structure for integrating patient safety, access and equity, communication, remote data collection, technology use, ethics, and systems-based care [10]. Platform-oriented learners need authentic cases in which they must balance a patient’s desire for rapid access with a clinician’s threshold for safe remote management. Simulation and supervised virtual encounters can require learners to obtain a focused history, identify what cannot be examined remotely, explain uncertainty, protect privacy, and negotiate escalation. Vietnamese educational research indicates that technology becomes meaningful when supported by faculty development, infrastructure, and purposeful curricular integration [28]. Evidence on interactive telemedicine indicates that effects vary by clinical condition, intended function, and implementation context [29]. Medical education research likewise supports structured teaching in appropriateness assessment, communication, clinical reasoning, confidentiality, equitable access, and supervised practice [30, 31].

Implementation implications extend beyond undergraduate education. Continuing professional development should address remote assessment, documentation, consent, data protection, and hybrid-care pathways. Vietnam’s provincial telehealth experience demonstrates the potential value of linking lower-level facilities with higher-level expertise [15]. Legal recognition of remote care [16] and the national list of eligible conditions [17] provide an enabling policy foundation. However, this survey did not collect facility-tier, hospital-workload, insurance-reimbursement, or out-of-pocket payment data, so it cannot determine how those factors shaped stakeholder perceptions. Routine implementation will still require technical support, clinical governance, clear referral processes, workforce participation, public trust, sustainable financing, and ongoing evaluation. Recent work on AI-assisted formative assessment offers a relevant educational parallel: digital tools add value only when users critically interpret outputs and preserve professional judgment, learner agency, privacy, and human accountability [32].

### Strengths and limitations

The main strength of this study is the inclusion of practicing doctors, patients or caregivers, and medical students within one analysis, supported by response-level recoding, transparent denominators, and an explicit coding matrix. Several limitations are important. First, convenience recruitment through electronic institutional channels creates self-selection and digital-literacy bias; people who were comfortable with online tools may have been more likely to participate. Invitation denominators and detailed nonresponse characteristics were not retained. Second, the qualitative extract did not retain detailed physician, patient, or student characteristics or whether the open-ended items were mandatory in each form version, limiting assessment of transferability and interpretation of completion differences. Third, the patient instrument at the pediatric site framed the benefit and appropriate-use questions around the child, so those responses are not fully equivalent to the doctor, student, and general-hospital versions. Fourth, stakeholder group was confounded with response language: doctors and patient or caregiver respondents answered in Vietnamese, whereas students answered in English. Lexical and academic-register differences may therefore have influenced the platform-versus-clinical contrast, which cannot be attributed solely to stakeholder role. Fifth, response length and the mean number of displayed domain assignments varied by group and question; longer or more elaborated responses had more opportunities to contribute multiple domains, so frequency differences may reflect elaboration as well as thematic salience. Sixth, the final domains were non-exhaustive, and a small number of meaningful responses remained unclassified. Seventh, short free-text responses provide breadth but less contextual depth than interviews or focus groups, and translation into English may have reduced nuance despite verification against the Vietnamese source. Finally, the study examined perceptions rather than observed practice, clinical outcomes, or actual national patterns of virtual healthcare use.

Future research should use purposive interviews and longitudinal or implementation-focused designs to examine how stakeholder expectations evolve and how education, regulation, workflow, and financing influence actual virtual care. Intervention studies should test whether stakeholder-informed curricula improve case selection, communication, privacy awareness, and escalation decisions.

## Conclusion

Doctors, patients or caregivers, and medical students shared a broad recognition of virtual healthcare but emphasized different aspects of it. Students had the highest proportion of platform-related descriptions and the lowest proportion of clinical-care descriptions, doctors emphasized consultation and clinical safety, and patients or caregivers more often invoked convenience and accessibility when defining virtual healthcare. Because response language and elaboration varied by group, these patterns should not be interpreted as pure stakeholder effects. They nevertheless support education and implementation strategies that connect technological fluency with clinical judgment and patient experience. Virtual healthcare curricula should include authentic case selection, remote communication, privacy and consent, explicit recognition of examination limits, and clear escalation to in-person care.

## Supplementary Information

Supplementary Material 1.

## Data Availability

The summarized coded data are included in the manuscript figures and figure legends. Raw free-text responses are not publicly available because they may contain potentially identifiable contextual information. Deidentified coded data and analytic materials are available from the corresponding author upon reasonable request and subject to institutional and ethical approval.

## Abbreviations

LMICs: Low- and middle-income countries
NPT: Normalization Process Theory
SRQR: Standards for Reporting Qualitative Research
