# Supplementary material for: Stakeholder perceptions of virtual healthcare in Vietnam: qualitative content analysis of doctors, patients, and medical students
Source: BMC Med Educ. 2026 Aug 18;26:1849. doi: 10.1186/s12909-026-10166-1 (PMC13760795; doi:10.1186/s12909-026-10166-1)
Supplement: Supplementary file 1 [file 12909_2026_10166_MOESM1_ESM.docx]

**Additional file 1**

Domain frequencies are respondent-level counts: each domain was recorded once per analyzable response, even when it was mentioned repeatedly. A response could contribute to multiple domains. The final domains summarized major recurring patterns and were not exhaustive; some meaningful responses did not map to a displayed domain. Percentages may therefore sum to more or less than 100%. D, doctors; P, patients/service users/caregivers; S, medical students. Quotation labels report respondent identifier, role, recruitment setting, and source language. Quotations translated from Vietnamese were checked against the source response.

**Table A1. Illustrative progression from source text to descriptive code and final domain**

| **Source text** | **Initial descriptive code** | **Final domain** | **Respondent** |
| --- | --- | --- | --- |
| “...without going directly to a hospital or clinic.” | Remote; no physical contact | Remote access without an in-person visit | DNCH128, doctor, public pediatric hospital; translated from Vietnamese |
| “...saves time, avoids waiting, and substantially reduces unnecessary costs.” | Time saving; cost saving; reduced travel | Cost, time, and travel saving | DNCH12, doctor, public pediatric hospital; translated from Vietnamese |
| “Diagnosis is also constrained because assessment is limited to questioning, listening, and visual observation.” | Lack of physical examination; diagnostic limitation | Clinical and quality limitations | DNCH12, doctor, public pediatric hospital; translated from Vietnamese |
| “...monitoring healthy people and patients with stable chronic disease who do not require emergency care...” | Chronic disease; follow-up and monitoring | Chronic disease management; follow-up and monitoring | DVMEC64, doctor, private general hospital; translated from Vietnamese |

**Table A2. Comprehensive qualitative coding matrix**

| **Question** | **Final domain** | **Constituent descriptive codes** | **Operational definition** | **D / P / S, n/N (%)** | **Exemplar quotation** |
| --- | --- | --- | --- | --- | --- |
| Q1 | Virtual consultation | Consultation; telemedicine-related terms | Remote advice or consultation between a patient and health professional; this domain captured the interactional mode rather than explicit diagnosis, treatment, prescribing, or monitoring | 214/370 (57.8%); 132/356 (37.1%); 74/213 (34.7%) | “Virtual healthcare means receiving online advice from doctors when needed, especially when a family member has a health problem and does not need to go to the hospital immediately.” [PVMEC91, patient/caregiver, private general hospital; translated from Vietnamese] |
| Q1 | Clinical care activities | Medical service; diagnosis; treatment; prescribing; monitoring | Explicit diagnosis, treatment, monitoring, prescribing, or another clinical service function; a response could also be coded as virtual consultation when both elements were present | 135/370 (36.5%); 121/356 (34.0%); 40/213 (18.8%) | “Virtual healthcare is consultation, diagnosis, and health monitoring delivered remotely through technology platforms such as websites, mobile applications, or video calls.” [DNCH128, doctor, public pediatric hospital; translated from Vietnamese] |
| Q1 | Digital platforms and technology | Online platform; application/device; AI technology; electronic record | Use of internet platforms, applications, devices, records, or AI to enable care | 114/370 (30.8%); 88/356 (24.7%); 76/213 (35.7%) | “...video consultations, remote monitoring, telemedicine, mobile health apps, and online health records... technologies like video conferencing, wearable health devices, and AI-driven health assessments.” [SVUNI83, medical student; original English] |
| Q1 | Remote access without an in-person visit | Remote; no physical contact | Care delivered across distance or without attendance or direct contact | 96/370 (25.9%); 90/356 (25.3%); 53/213 (24.9%) | “Remote examination and treatment use internet-based tools so that the patient does not need to meet the doctor in person.” [DVMEC64, doctor, private general hospital; translated from Vietnamese] |
| Q1 | Video-based communication | Video call; synchronous audiovisual contact | Explicit reference to video-mediated communication | 57/370 (15.4%); 56/356 (15.7%); 37/213 (17.4%) | “Examination and consultation without direct contact, using only video calls.” [DNCH1, doctor, public pediatric hospital; translated from Vietnamese] |
| Q1 | Convenience and accessibility | Accessibility; convenience; ease; privacy | Ease, speed, privacy, or ability to obtain care | 46/370 (12.4%); 71/356 (19.9%); 12/213 (5.6%) | “It allows people to access medical services comfortably and privately.” [PNCH82, patient/caregiver, public pediatric hospital; translated from Vietnamese] |
| Q2 | Cost, time, and travel saving | Cost saving; time saving; reduced travel | Reduced direct or indirect cost, waiting time, travel, or work disruption | 297/370 (80.3%); 261/352 (74.1%); 141/213 (66.2%) | “It is easy to access remotely, saves time, avoids waiting, and substantially reduces unnecessary costs.” [DNCH12, doctor, public pediatric hospital; translated from Vietnamese] |
| Q2 | Convenience and accessibility | Convenience; flexibility; accessibility; specialist reach | Flexible or easier access, including for remote or underserved populations | 217/370 (58.6%); 139/352 (39.5%); 130/213 (61.0%) | “Living in an urban area, virtual health for me was very convenient and saved time as I would not have to travel and queue up in these crowded clinics or hospitals... it enhances access to specialists who may not be available locally.” [SVUNI149, medical student; original English] |
| Q2 | System efficiency, continuity, or safety | Monitoring; reduced infection; reduced hospital overload; coordination | Continuity, remote monitoring, reduced crowding or infection exposure, or coordination | 65/370 (17.6%); 43/352 (12.2%); 48/213 (22.5%) | “Virtual healthcare can reduce hospital overcrowding and save patients time and travel costs.” [DNCH109, doctor, public pediatric hospital; translated from Vietnamese] |
| Q3 | Clinical and quality limitations | Lack of physical examination; diagnostic challenge; information limitation; emergency limitation | Inability to examine directly, obtain sufficient information, diagnose confidently, or manage urgent conditions | 203/368 (55.2%); 124/341 (36.4%); 78/206 (37.9%) | “Diagnosis is also constrained because assessment is limited to questioning, listening, and visual observation.” [DNCH12, doctor, public pediatric hospital; translated from Vietnamese] |
| Q3 | Technical and workforce barriers | Connectivity; hardware/software; interoperability; training/workforce | Technology, infrastructure, integration, or workforce capability limitations | 141/368 (38.3%); 120/341 (35.2%); 113/206 (54.9%) | “The implementation of virtual healthcare faces several barriers, including limited access to reliable internet and digital devices, particularly in rural or low-income areas... some patients and healthcare providers may lack digital literacy or be resistant to technology-based care.” [SVUNI83, medical student; original English] |
| Q3 | Access and population-related barriers | Digital literacy; limited accessibility; finance; insurance | Digital skills, affordability, insurance, or population access constraints | 82/368 (22.3%); 51/341 (15.0%); 50/206 (24.3%) | “...many patients, especially older ones, are not familiar with using technology, creating a digital literacy gap.” [SVUNI149, medical student; original English] |
| Q3 | Trust, privacy, and regulatory barriers | Lack of trust; privacy/legal issues; regulatory uncertainty; cultural resistance | Doubts about accuracy, confidentiality, legal clarity, or preference for face-to-face care | 65/368 (17.7%); 44/341 (12.9%); 50/206 (24.3%) | “I worry about scams... I would feel more reassured if the service were provided by a hospital... I still feel safer with an in-person examination.” [PVMEC25, patient/caregiver, private general hospital; translated from Vietnamese] |
| Q4 | Common and non-urgent conditions | Common illness; minor/stable condition; non-emergency condition | Minor, familiar, stable, or non-emergency health problems | 92/367 (25.1%); 136/355 (38.3%); 86/205 (42.0%) | “When a child has a mild cough or a minor oral problem, medication may be prescribed without complex tests.” [PNCH65, patient/caregiver, public pediatric hospital; translated from Vietnamese] |
| Q4 | General advice and consultation | Initial advice; orientation; health education; referral guidance | Initial advice, education, orientation, or support deciding when and where to seek care | 151/367 (41.1%); 85/355 (23.9%); 38/205 (18.5%) | “General health advice, initial management when illness is suspected, and guidance on monitoring and when hospital attendance is needed.” [DVMEC37, doctor, private general hospital; translated from Vietnamese] |
| Q4 | Mental health and psychological care | Mental health; counseling; therapy; psychiatric review | Counseling, therapy, psychological support, or psychiatric consultation | 64/367 (17.4%); 10/355 (2.8%); 63/205 (30.7%) | “Virtual healthcare is ideal for addressing non-emergency issues like routine check-ups, mental health counseling, prescription refills, chronic disease management, and minor illnesses...” [SVUNI128, medical student; original English] |
| Q4 | Dermatology and skin conditions | Dermatology; skin; image-supported review | Skin conditions considered suitable for visual review or advice | 53/367 (14.4%); 28/355 (7.9%); 43/205 (21.0%) | “Rashes, eczema, and acne.” [SVUNI41, medical student; original English] |
| Q4 | Follow-up and monitoring | Follow-up; post-treatment review; result discussion; periodic check | Post-treatment review, monitoring, result discussion, or periodic assessment | 52/367 (14.2%); 32/355 (9.0%); 36/205 (17.6%) | “Initial health advice, psychological counseling, and follow-up after treatment for conditions that do not require investigations.” [DVMEC59, doctor, private general hospital; translated from Vietnamese] |
| Q4 | Chronic disease management | Chronic disease; medication adjustment; long-term management | Ongoing management of diabetes, hypertension, asthma, or other chronic illness | 76/367 (20.7%); 11/355 (3.1%); 28/205 (13.7%) | “...monitoring healthy people and patients with stable chronic disease who do not require emergency care...” [DVMEC64, doctor, private general hospital; translated from Vietnamese] |
| Q4 | Nutrition and lifestyle issues | Nutrition; diet; supplements; exercise; preventive lifestyle | Nutrition, supplementation, lifestyle, exercise, or preventive advice | 35/367 (9.5%); 29/355 (8.2%); 9/205 (4.4%) | “Advice about nutrition, micronutrient supplementation, and common mild symptoms in children.” [PNCH122, patient/caregiver, public pediatric hospital; translated from Vietnamese] |
| Q4 | Reproductive or condition-specific care | Reproductive health; pregnancy; fertility; other specific care | Reproductive, obstetric, fertility, or other explicitly condition-specific consultation | 16/367 (4.4%); 15/355 (4.2%); 10/205 (4.9%) | “Advice on pregnancy planning and methods to support conception.” [DVMEC122, doctor, private general hospital; translated from Vietnamese] |

Note: Percentages use the analyzable denominator for the corresponding question and stakeholder group. The patient group included patients, service users, and caregivers. Question 2 and question 4 were child-referenced for patient or caregiver respondents recruited at the pediatric hospital. Caregivers at the general hospital could also frame unrestricted responses around a child. The displayed domains were non-exhaustive; aggregate assignments imply that at least two patient or caregiver question 3 responses and at least nine question 4 responses did not map to a displayed domain. Because other responses could map to multiple domains, aggregate totals do not identify the exact number of unclassified responses.

**Table A3. Mean number of displayed domain assignments per analyzable response**

| **Question** | **Doctors** | **Patients/service users/caregivers** | **Medical students** |
| --- | --- | --- | --- |
| Q1: Meaning | 1.79 | 1.57 | 1.37 |
| Q2: Benefits | 1.56 | 1.26 | 1.50 |
| Q3: Barriers | 1.33 | 0.99 | 1.41 |
| Q4: Appropriate uses | 1.47 | 0.97 | 1.53 |

Note: Values equal the sum of respondent-level displayed-domain assignments divided by the analyzable denominator for each stakeholder-question cell. They contextualize differences in response elaboration; longer or more detailed responses had more opportunities to contribute multiple domains. Values below 1.00 also reflect the non-exhaustive final domain set.
